# Distractor-Aware Deep Regression for Visual Tracking

**DOI:** 10.3390/s19020387

**Published:** 2019-01-18

**Authors:** Ming Du, Yan Ding, Xiuyun Meng, Hua-Liang Wei, Yifan Zhao

**Affiliations:** 1 Key Laboratory of Dynamics and Control of Flight Vehicle, Ministry of Education, School of Aerospace Engineering, Beijing Institute of Technology, Beijing 100081, China; dmpyz09@gmail.com (M.D.); mengxy@bit.edu.cn (X.M.); 2 Department of Automatic Control and Systems Engineering, University of Sheffield, Sheffield S1 3JD, UK; w.hualiang@sheffield.ac.uk; 3 Through-Life Engineering Services Institute, School of Aerospace, Transport and Manufacturing, Cranfield University, Cranfield, Bedfordshire MK43 0AL, UK; yifan.zhao@cranfield.ac.uk

**Keywords:** object tracking, deep-regression networks, data imbalance, distractor aware

## Abstract

In recent years, regression trackers have drawn increasing attention in the visual-object tracking community due to their favorable performance and easy implementation. The tracker algorithms directly learn mapping from dense samples around the target object to Gaussian-like soft labels. However, in many real applications, when applied to test data, the extreme imbalanced distribution of training samples usually hinders the robustness and accuracy of regression trackers. In this paper, we propose a novel effective distractor-aware loss function to balance this issue by highlighting the significant domain and by severely penalizing the pure background. In addition, we introduce a full differentiable hierarchy-normalized concatenation connection to exploit abstractions across multiple convolutional layers. Extensive experiments were conducted on five challenging benchmark-tracking datasets, that is, OTB-13, OTB-15, TC-128, UAV-123, and VOT17. The experimental results are promising and show that the proposed tracker performs much better than nearly all the compared state-of-the-art approaches.

## 1. Introduction

Visual-object tracking aims to estimate the trajectory of the specified target in a video sequence, which is labeled in the initial frame with a bounding box. It is widely used in various applications, ranging from video surveillance, motion analysis, and autonomous driving. The main difficulty of visual tracking is how to accurately and effectively locate the object in challenging scenarios caused by illumination, deformation, occlusion, out of view, background cluttering, and other variations [1,2,3].

Modern tracking methods can be classified as either generative or discriminative. Generative methods aim at describing the target appearance using some generative processes (e.g., statistical models [4], templates [5], or sparse coding [6,7,8,9,10]), and searching the candidates to minimize reconstruction errors. Discriminative approaches instead regard tracking as a classification problem by differentiating target appearance and the surrounding background. Numerous classifiers have been introduced in the tracking community, such as structured support vector machine [11,12], boosting [13], oblique random forests [14], and online multiple-instance learning [15]. Recently, discriminative correlation filters (DCF) trackers [16,17,18,19,20,21,22,23,24,25], which directly regress the dense samples to soft labels, have sparked significant attention due to their high accuracy and efficiency. Furthermore, the favorable performance of deep convolutional networks on several challenging vision tasks [26,27,28] encourages recent works to either exploit existing deep convolutional-neural-network (CNN) features within discriminative correlation filters [29,30,31,32,33], or design deep architectures [34,35,36,37,38,39,40] for discriminative visual tracking.

Despite top performance on benchmarks, discriminative correlation filters take few advantages of end-to-end training. On the contrary, deep regression networks that reformulate the correlation operation as a convolutional layer are fully differentiable and can be trained end to end. In this paper, we show that a deep regression tracker, when properly designed, can achieve even better and competitive results compared with the state-of-the-art trackers, especially with discriminative correlation filters. A critical issue relating to deep regression trackers is the data-imbalance problem [39,41,42] where the number of samples in the negative class is large but it is much smaller in the positive class. It is known that “easy” negative samples dominate the training dataset, and this inevitably suppresses the contribution of positive samples and therefore affects the performance of the resulting regression networks. In addition, distractor samples whose semantic abstraction close to the target may also be suppressed despite having an important contribution to model robustness. The existing approaches to dealing with data imbalance in regression trackers mainly focus on reducing the role and influence of background samples and pay little attention to the contribution of semantic distractors. In this work, we try to address the imbalance-distribution issue by introducing a novel distractor-aware loss function during the learning of the regression networks. The proposed distractor-aware loss function differs from existing approaches (e.g., shrinkage loss [39] and focal loss [43]) in that our approach barely utilizes easy negative samples and increase the importance of distractor samples (see Section 3.2), whereas shrinkage loss only penalizes easy samples and focal loss partially reduces the importance of hard samples.

To further promote the performance of deep feature-based trackers, many efforts have been made in the visual-object tracking communities. Leveraging multilevel semantic abstraction exploitation across multiple convolutional layers has drawn more and more attention. Residual connections across multiple convolutional layers can mine distinguished information compared with independently learning on multiple convolutional layers. However, residual connected regression networks will become massive when the across-number of convolutional layers increases. Furthermore, the lack of training data often makes the regression networks unable to effectively balance the numerical values of multi-layer features. In this work, we propose to apply hierarchy-normalized concatenation to fuse multiple convolutional layers. The proposed scheme, with a simple structure and without numerical issue, can make full use of multi-level convolutional features across multiple convolutional layers. By taking advantage of distractor-aware loss and hierarchy-normalized concatenation, the proposed tracking method achieves favorable results against the state-of-the-art trackers on several tracking benchmarks.

The main contributions of this work can be summarized as three parts:

1. The proposed novel distractor-aware loss can alleviate the data-imbalance issue in learning deep-regression networks. We observe that the adversarial semantic distractors not only facilitate robustness in the tracking phrase but also accelerate convergence in the training phrase.
2. We leveraged hierarchy-normalized concatenation to fully exploit multilevel semantic abstraction across multiple convolutional layers. This results in a simple and easy-to-train end-to-end regression network for visual tracking.
3. We extensively validated the proposed method on five benchmark datasets with large-scale sequences. The proposed tracking algorithm had favorable results compared with state-of-the-art trackers on all benchmark datasets. Furthermore, as far as we know, it achieves leading performance in both OTB-13 [1] and OTB-15 [2]. To facilitate further studies, our source code, as well as all experimental results, are available at https://github.com/Dewly/DaDRT.

## 2. Related Work

Visual tracking has long been an active research topic with extensive surveys and benchmark evaluations. In this section, we give a brief review on most relevant tracking approaches and pay attention to the data-imbalance issue. Comprehensive reviews on object tracking can be found in References [2,44,45].

### 2.1. Trackers with Correlation Filters

Correlation filters have recently attracted considerable attention in the object-tracking community due to their high computational efficiency and favorable robustness. Trackers based on correlation filters regress all circular-shifted versions of input samples to Gaussian-like soft labels. Bolme et al. [46] proposed to exploit the correlation filters for visual tracking and optimized the output sum of squared errors in the Fourier domain, leading to an extremely fast tracker at 669 frames per second (FPS). Henriques et al. [16] first suggested to regress all circular shifted versions of illumination-intensity features to a Gaussian label. Furthermore, Henriques et al. [17] incorporated kernel functions into the correlation filter and extended the correlation filters to multiple channel histograms of gradient (HOG) features. Inspired by the work of Reference [16], several extensions have been proposed to promote the accuracy and robustness of correlation filter-based trackers. Extensions include, but are not limited to, kernelized correlation filters [17,47], scale estimation [19,48], redetection [24], spatial regularization [20,21,22,31], context learning [23,49] and CNN feature integrations [29,32,33,50].

### 2.2. Trackers with Deep Regression

Benefiting from the strong representation of CNN features, correlation filter-based trackers have achieved distinguishable performance. However, associated optimization in Fourier domain suffers the boundary effect. Different from traditional correlation filter-based trackers, deep-regression trackers try to obtain an approximate solution via gradient descent in spatial domain. They formulate the correlation filter as a convolution operation and build a one-channel-output convolution layer, as used in a typical convolutional neural network. Recent trackers [35,38,39,41] follow this manner and achieve significant improvement in performance on par with correlation-filter trackers. Chen et al. [41] introduced a single-layer regression model for visual tracking and exploited a novel automatic hard-negative mining method to facilitate training the regression model. Wang et al. [35] introduced a fully convolutional network to exploit multiple CNN features by leveraging a feature-map selection strategy. Both a top layer and a lower layer were jointly used with a switch mechanism during tracking. Song et al. [38] proposed to apply residual learning to take appearance changes into account on a single convolutional layer, and formulated the tracking progress in an end-to-end manner by integrating feature extraction, response-map generation, and model updates into the neural networks. Lu et al. [39] proposed to apply residual connections to fuse two convolutional layers as well as their output response maps, achieving remarkable results. However, we observed that residual connections across multiple layers are more or less suffering from the numerical-imbalance issue (see Section 3.3). In order to tackle the numerical-imbalance issue, we propose to apply a hierarchy-normalized concatenation operation to directly connect multiple convolutional layers. The novel connection is fully differential and can make the network more concise.

### 2.3. Data Imbalance

The data-imbalance issue often exists in learning applications and has been extensively studied. Hard-negative mining [51,52], data resampling [53,54], and cost-sensitive loss [55,56] are helpful to alleviate the data-imbalance issue to some extent. In visual tracking, it is possible to incorporate vast samples from the whole image. However, the numbers of positive samples are extremely limited and far fewer than the number of negative samples. Imbalance distribution between positive and negative deteriorates the performance of the trackers. Chen et al. [41] applied the automatic hard-negative mining method to eliminate easy negatives and enhance positives. A recent work [43] on dense-object detection proposed focal loss to decrease loss from easy samples as well as partially decrease the loss of hard samples. Song et al. [42] proposed to apply cost-sensitive loss to decrease the effect from easy negatives. Lu et al. [39] exploited shrinkage loss to penalize the loss from easy samples, and kept the hard samples unchanged. Unlike the aforementioned solutions for the data-imbalance issue, we propose a distractor-aware loss to adaptively reinforce significant semantics and extremely penalize a pure background. This scheme improves both tracking accuracy and robustness, and accelerates training convergence.

## 3. Proposed Algorithm

**Figure 1:**
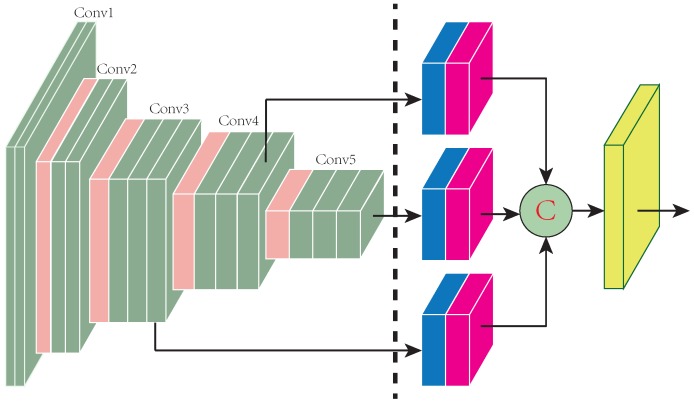
Overview of the proposed deep regression network. The left-side blocks are the fixed-feature extractor backbone. The right-side blocks are trained in the first frame and updated frame by frame. The blue blocks represent the 1 × 1 channel reduction layers, and red blocks represent the normalization and rescale layers. The yellow block indicates the target-specified one-channel-output convolutional regression layer. The connection circle means hierarchy concatenation. The proposed network effectively exploits semantic abstraction across three convolutional layers.

The proposed Distractor-aware Deep Regression Tracking (DaDRT) algorithm follows a general one-channel-output regression framework. In addition, we exploit a novel distractor-aware loss function to handle data imbalance and introduce a hierarchy-normalized concatenation connection to fully exploit multilevel semantics across convolutional layers. Figure 1 shows an overview of our pipeline. Details are discussed below.

### 3.1. Regression via Convolution Layer

The regression model for visual tracking aims to regress dense samples to Gaussian-like soft labels. Here, we revisit the linear-ridge regression model and formulate the regression model as one convolutional layer. Given an initial image with labeled target, we can extract dense sample features **X**, and generate corresponding Gaussian function labels **Y**. The coefficients **W** for regression function f=X∗W are estimated by solving the following minimization problem:

(1) $$\min_{W}{\{∥W*X-Y∥}_{2}^{2}+{\lambda ∥W∥}_{2}^{2}\}$$

where ∗ denotes the convolution operation, and λ is a regularization parameter that controls overfitting. Particularly, there exists a closed-form solution for this problem by transforming the convolution operation between coefficients **W** and samples **X** into an elementwise product in the Fourier domain. Here, we try to solve the regression problem in the spatial domain by reformulating the problem as loss minimization of the convolutional neural network.

The loss function in Equation (1) can be reinterpreted as:

(2) $$L(W)={∥F\left(W,X\right)-Y∥}_{2}^{2}+\lambda {∥W∥}_{2}^{2}$$

where F(W,X)=W∗X is the network output, **W** is the network weights, and **Y** is the ground-truth labels. The convolution operation on **X** can be carried out via a convolution layer with one-channel output. The size of the convolution kernel in the regression layer is different from conventional convolution layers that adopt a small fixed receptive field, such as 3 × 3 and 5 × 5. We set the receptive-field size of the regression layer to the size of a tracked target in our framework. The convolutional weights can be effectively calculated by iteratively optimizing **W** in Equation (2) using the gradient descent method, which can be implemented in almost all modern deep-learning frameworks. Regularization parameter λ is usually explained as weight decay in deep-learning frameworks. In this study, the value of parameter λ is determined by following the default setting in the implemental platform.

### 3.2. Distractor-Aware Loss

**Figure 2:**
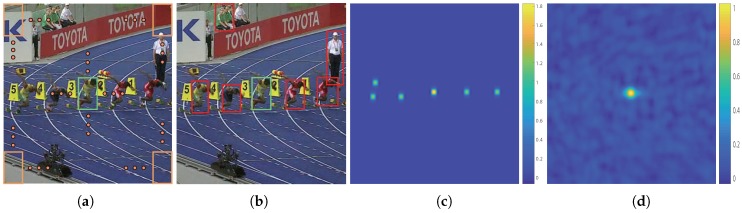
(**a**) Sliding sampled region of interest (ROI) centered on the target. (**b**) Obvious semantic distractors labeled in red bounding boxes. (**c**) Modulating factor in one training epoch. Note that the easy background is extremely suppressed, and the target and several selected distractors are reinforced. (**d**) Output regression map of the regression network.

For deep-regression tracking, it is possible to exploit all real extracted samples by sliding a window fashion over the whole image during the training and detecting stages. The region of interest (ROI) contains a large amount of context surrounding the target object, as shown in Figure 2a. The large surrounding context contains a majority of pure background and few semantic distractors, as shown in Figure 2b. A large amount of background helps strengthen the discriminative power of the target object from the background, and distractors help discriminate the target object from a similar context. However, this also leads to an increase in the number of easy negative samples. Reviewing Equation (2), when summed over the large input search area, the loss values from easy negative samples submerge the valuable and rare positive samples and distractors. The learning progress usually drifts to a classification problem between objects and backgrounds due to the easy negative samples dominating the gradient. The tracker is less robust to similar semantic distractors.

Existing solutions to the data-imbalance issue mainly focus on penalizing the importance of easy negative samples. However, we observed that semantic distractors have a significant contribution in learning deep-regression networks. We propose to add a modulating factor to the loss that highly suppresses pure backgrounds and protrudes the target and semantic distractors, as shown in Figure 2c. Formally, we formulate our distractor-aware loss function as:

(3) $$L(W)={D\;⊙∥F\left(W,X\right)-Y∥}_{2}^{2}+\lambda {∥W∥}_{2}^{2}$$

where **D** is a distractor-aware modulating factor that balances training sample loss, and ⊙ denotes the elementwise product. In this work, we adaptively carried out the modulating factor for each optimization iteration. We denote the regression network output in every iteration by **R**, which generally indicates the probability of a position to be the target object. Once we obtained the probability map, we first identitied a number of **N** semantic objects by locating the local maximum of **R**. In general, there is always one positive sample at the map center and **N** minus one distributed negative distract samples. For each semantic sample, we generated a basic modulator **D**_*b*_ in terms of soft labels **Y** centered at the identified positions:

(4) $$D_{b}=Y⊙e^{k(Y-1)}$$

where *k* is a scalar penalization factor. Modulator **D**_*b*_ aims at highlighting the central influence and suppressing the surrounding influence. Then, we merged the **N** basic modulators by summation. In addition, we took one more modulator on the target location to increase the importance of positive samples. Figure 2c illustrates a modulating factor in the training iteration. Note that, in comparison with general deep-regression trackers, the proposed loss function introduces two extra hyperparameters, that is, *k* and **N**. We observed that the larger the value of *k* was, the larger the penalty to the surrounding contexts. A larger **N** means taking more distractors to take into account, which may overwhelm the only positive sample. On the other hand, a smaller **N** makes the training process overfit to the target. Considering the proportion of positive and distractor samples, we fixed the k=1.6 and the number of semantic objects **N** to be 6 in all our experiments, meaning we introduced five adversarial distractors to go against the positive sample.

By applying the modulating factor, distractor-aware loss mainly focuses on positive samples and adversarial distractors. Extensive comparison with other losses shows that our distractor-aware loss not only promotes tracking accuracy but also accelerates training speed (see Section 5.3).

### 3.3. Hierarchy-Normalized Concatenation

**Figure 3:**
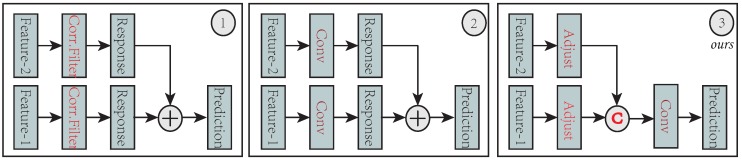
Three types of feature connection. (1) Independent connection that is used in traditional correlation-filter trackers (e.g., ECO [32]). Pediction is obtained by weighted summing from different correlation-filter responses. (2) By substituting a traditional correlation filter with a convolutional layer, the second type can exploit various end-to-end residual connections. CREST [38] exploits only a single convolutional layer (*conv4_3* from *VGG16*) that means *feature-1* and *feature-2* are the same to form a base and a residual network. The DSLT [39] leverages two convolutional layers (*conv4_3* and *conv5_3* from *VGG16*) to learn the residual across multiple convolutional layers. (3) Our scheme exploits the feature adjuster (normalization) to fuse multiple convolutional layers. We learn a regression layer directly on the reinforced feature represented. The proposed scheme can easily incorporate more features without increasing the convolutional parameters.

The convolutional layers of a typical CNN model, e.g., VGG [28], provide different levels of semantic abstraction in the features hierarchies. Features in the earlier layers, with higher spatial details, are helpful in precise localization; features in the later layers capture more semantic abstraction and are robust to large appearance changes. Motivated by this observation, many efforts have been made to exploit the merits of multiple convolutional layers, including independent learning [32,35] and residual connection [38,39]. Danelljan et al. [32] proposed to independently learns correlation filters over the *conv1* and *conv5* layers and merges the corresponding output response maps with empirical weights. Wang et al. [35] proposed to independently exploit two branch networks on the *conv4* and *conv5* layers, and merge the output response map with a distractor-detection scheme. Song et al. [38] proposed to apply residual connections on a single *conv4* layer to capture the difference between the base layer output and the ground truth. Lu et al. [39] exploited the residual connections to fuse *conv4* and *conv5* layers as well as their output response maps. Here, we propose a novel hierarchy-normalized concatenation connection to make full use of multiple-level semantic abstraction. We introduced a normalize operation to balance the features’ numeric value and then concatenated the *conv3*, *conv4*, and *conv5* layers to form a strong representation for visual tracking. We compare our scheme with the aforementioned connections in Figure 3.

**Figure 4:**
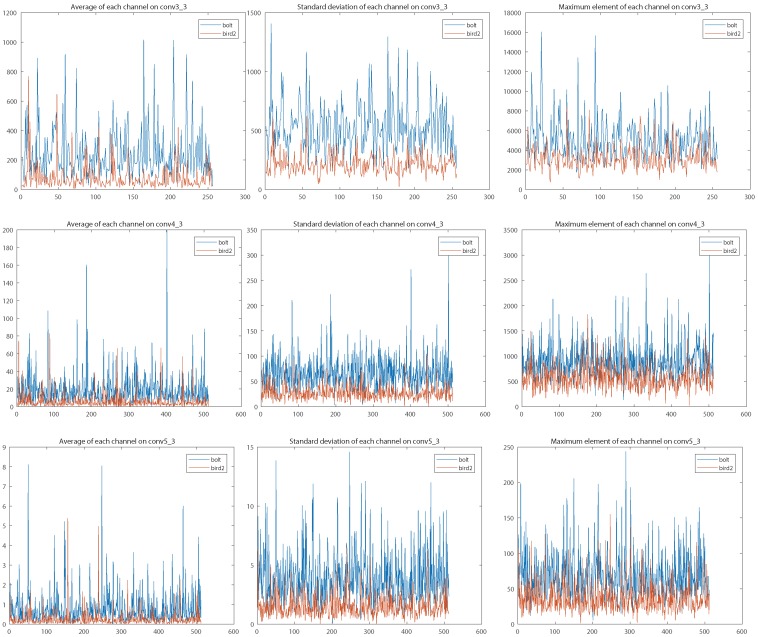
Visualizations of numeric distribution at different abstraction levels on two sequences. High-level abstractions (**bottom** row) emerge with smaller values compared with low-level abstractions (**top** row). It is unreasonable to directly add the features of different convolutional layers together. The limited training samples and epochs cannot afford enough learning procedure to obtain the appropriate model parameters. Results are best viewed on high-resolution displays.

Different-level layers represent different types of semantic abstraction. We observed that integrating across multiple convolutional layers usually suffers from the numeric issue, in which different layers have significant divergence numeric distribution. We illustrate the numerical statistics of features from three *VGG16* convolutional layers in Figure 4. In general, the regression learning progress cannot afford enough epochs and samples to regulate the convolutional weights of each connection branch due to fast convergence. The training process leans to one branch because of numeric magnitude rather than discrimination power. An existing solution for alleviating the dilemma is to adjust the convolutional weights and learning rates during the network initialize phase. However, fixed initialization does not harmonize with diversiform tracking sequences. In order to tackle the numeric issue, we propose to apply L2 normalization (the red blocks in Figure 1) along the depth dimension for each semantic abstraction. In addition, we leverage a channel attention scheme to reweight the importance of feature channels. Our connection scheme not only jumps out the weight-initialization straits but also permits a higher learning rate in regression training, which accelerates convergence.

## 4. Tracking via DaDRT

We illustrate the detailed procedure of DaDRT for visual tracking. We decomposed our tracking process into four stages, namely, model initialization, online detection, scale estimation, and model update. Details are as follows:

- **Model Initialization**. At this stage, we follow the general tracking initialization process [17,32,38] to locate the target of interest as suggested by the benchmark [1,2,3,57,58]. In initial frame $I_{0}$, the target state is usually given by a bounding box $bb_{0}=\left\{x_{0},y_{0},w_{0},h_{0}\right\}$, where $\left\{x_{0},y_{0}\right\}$ denotes the left-top pixel position and $\left\{w_{0},h_{0}\right\}$ indicates the target width and height respectively. We leveraged a new bounding box $cp_{0}=\left\{x_{0}+\left(1-c_{x}\right)w_{0}/2,\;y_{0}+\left(1-c_{y}\right)h_{0}/2,\;c_{x}w_{0},\;c_{y}h_{0}\right\}$ to crop the sample patch for tracking initialization, where scalars $c_{x}$ and $c_{y}$ denote amplification factors; in this study, we suggest $c_{x}=5,c_{y}=9$. Especially for the unbalanced target-aspect ratio, we fixed the amplification factor of the long side to 5, and a larger amplification to the short side to keep the bounding box squarelike. Once the sample patch is acquired, we adopt the tailored *VGG16* network as the backbone-feature extractor and feed the sample patch into the extractor. Then, we take the output of the *conv3_3*, *conv4_3*, and *conv5_3* layers as deep features for further training the regression network. The data flow is illustrated in Figure 1. Meanwhile, all parameters in the regression layers are randomly initialized following the improved Xavier [59] method. The regression layers are well-initialized after a number of training steps.
- **Online Detection**. For current frame $I_{t}$, the previous predicted target state $bb_{t-1}=\left\{x_{t-1},y_{t-1},w_{t-1},h_{t-1}\right\}$ is utilized to derive the search patch bounding box $cp_{t}=\left\{x_{t-1}+\left(1-c_{x}\right)w_{t-1}/2,\;y_{t-1}+\left(1-c_{y}\right)h_{t-1}/2,\;c_{x}w_{t-1},\;c_{y}h_{t-1}\right\}$. The search patch is cropped according to bounding box $cp_{t}$ and is delivered to the designed network to generate a response map. Motion constraint is further introduced to increase the robustness. We leverage an isotropy Gaussian function to produce motion constraint map that penalizes large deviation away from the previous target location. We carry out the prediction map by elementwise multiplying the motion map with the response map. Once we obtain the prediction map, we predict the target object by locating the maximum prediction value.
- **Scale Estimation**. After obtaining the target position in the current frame, we extract scale search patches following the scale pyramid scheme as in ACF [48]. We generate the scale response map by feeding these scale search patches into our regression network. The index of maximum response indicates the current scale location. Then, we update the target scale by a smooth manner:
  (5) $$\left(w_{t},h_{t}\right)=\beta \left(w^{p}*,h^{p}\right)+\left(1-\beta \right)\left(w_{t-1},h_{t-1}\right)$$
  where $w_{t}$ and $h_{t}$ represent the width and height of the target object at frame *t*, respectively; and $w^{p}$, $h^{p}$ are the predicted width and height from the detection scheme. Scalar weight β enables a smooth update of the target scale.
- **Model Update**. In order to accommodate the model to the varied object appearance, we incrementally update our tracker frame by frame. For each frame, we crop the training patch relying on the estimated location and scale and generate corresponding soft labels. To alleviate model drift from noisy updates, training data pairs from past *T* frames are all adopted for online update.

## 5. Experiments

In this section, we introduce the implementation details and compare our DaDRT tracker with state-of-the-art trackers on five frequently used benchmark datasets for performance evaluation. Then, we conduct extensive ablation studies to analyze the effects of distractor-aware loss and hierarchy-normalized concatenation connection.

### 5.1. Implementation Setup

We implement the proposed DaDRT in Matlab using the matconvnet toolbox [60]. Our backbone-feature extractor is based on *VGG16* with only the first two pooling layers retrained. We extract the feature abstractions from the *conv3_3*, *conv4_3* and *conv5_3* layers; then, we apply a 1 × 1 convolutional layer to reduce the feature channels to 48. Regression labels and the motion map are both generated using a two-dimensional Gaussian function with a peak value of 1.0. We set the kernel width to be proportional (0.08) to the target size for the regression labels and proportional (1.0) to the geometric mean of target size for motion constraint map. In the initial training stage, we iteratively apply the SGD optimizer to update the weights in the regression network with a fixed learning rate of $8\;\times \;10^{-5}$ and weight decay of $5\;\times \;10^{-4}$, until the loss in Equation (3) is below a given threshold of 0.1, or the maximum 30 train epochs that are allowed are reached. In the updating stage, we adopt the training data pair from the past T=4 frames to update the network beyond two train epochs with a lower leaning rate $3\;\times \;10^{-5}$. For scale estimation, we utilize three levels of scale pyramid with the change ratio 5% and set the smooth update factor β=0.6. The optimal hyperparameters (e.g., learning rate and weight decay) are determined by the grid search method on a subset of OTB-15. Once the optimal hyperparameters are obtained, we fix the optimal hyperparameters for all the evaluation experiments. Our DaDRT tracker runs on a PC with an i7-2.4GHz CPU and a NVIDIA 1080-Ti GPU and the average speed is about 3 FPS. The source code and evaluation results will be publicly available.

### 5.2. State-of-the-Art Comparison

Here, we extensively evaluate the proposed DaDRT algorithm on five challenging benchmark datasets, OTB-13 [1], OTB-15 [2], TC128 [57], UAV123 [3], and VOT17 [58]. We follow the standard evaluation approaches and compare our results with other state-of-the-art trackers using the author-provided results for fair comparison. We used the same tracker configuration for all experiments.

#### 5.2.1. Comparison with OTB

There are two versions of OTB datasets, OTB-13 and OTB-15. OTB-15 is an extension of OTB-13, and the two datasets contain 50 and 100 challenging sequences, respectively. All sequences are labeled with ground-truth bounding boxes and various attributes including illumination variation (IV), scale variation (SV), occlusion (OCC), deformation (DEF), motion blur (MB), fast motion (FM), in-plane rotation (IPR), out-of-plane rotation (OPR), out-of-view (OV), background clutters (BC), and low resolution (LR). We compared our tracker with 29 trackers from the OTB benchmark and 39 other state-of-the-art trackers, including C-COT [31], CF2 [29], CREST [38], DCFNet [61], SRDCF [22], DeepSRDCF [30], DSST [19], ECO [32], HDT [50], deepLCT [62], LCT [24], MCPF [63], MDNet [36], SRDCFdecon [64], STAPLE [25], VITAL [42], PSCF [65], CNN-SVM [66], KCF [17], MEEM [67], MUSTer [68], HCF [69], ADNet [70], DLT [34], STC [71], TGPR [72], DSLT [39], BACF [21], DAT [73], PTAV [74], SiamRPN [75], DaSiamRPN [76], DLSSVM [77], BIT [78], FCNT [35], ACFN [79], and RCF [80]. We evaluated all tracker datasets using one-pass evaluation (OPE) with precision and success plots metrics as proposed in References [1,2]. The precision metric measures the frame-location rate within a certain threshold distance from ground-truth locations. Threshold distance was set as 20 pixels. The success plot metric was set to measure the overlap ratio between the predicted bounding boxes and the groundtruth.

**Figure 5:**
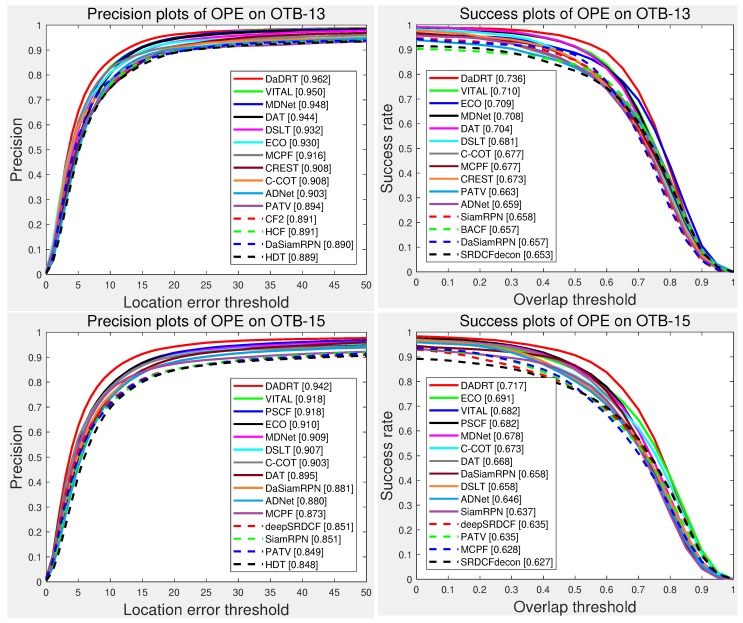
Precision and success plots using one-pass evaluation on the OTB-13 and OTB-15 datasets. The performance score for each tracker is shown in the legend. Our tracker achieves leading performance among the evaluated trackers. Results are best viewed on high-resolution displays.

Figure 5 shows the evaluation results with one-pass evaluation. We only show the top 15 trackers for presentation clarity. The distance-precision (DP) and area-under-curve (AUC) scores for each tracker are reported in the figure legend. The proposed DaDRT approach outperformed all the other trackers in terms of precision and success scores. Our tracker achieved leading performance with DP of 0.962/0.942 and AUC of 0.736/0.717 on OTB-13 and OTB-15, respectively.

**Figure 6:**
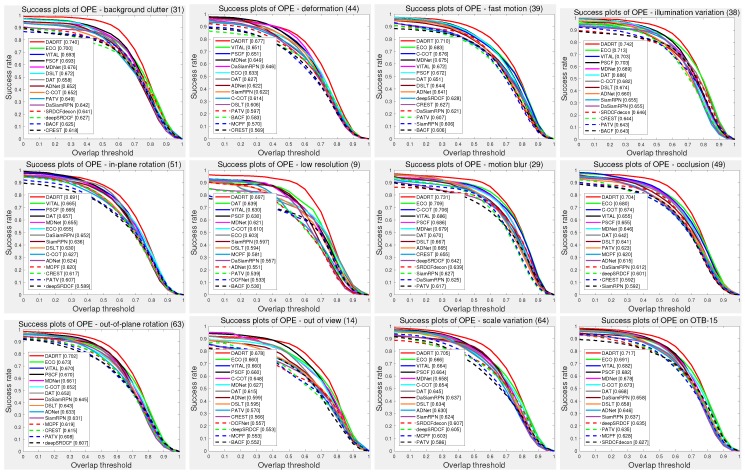
Overlap success plots under the eleven annotated video attributes. We only show the top 10 trackers for each challenging attribution. Our tracker ranked first in all attributes. Results are best viewed on high-resolution displays.

In addition, we further evaluated tracker performance under different video attributes on the OTB-15 dataset. Figure 6 compares performance under eleven annotated video attributes using one-pass evaluation with the AUC score. The results indicate that our DaDRT tracker is effective in handing all challenging attributes, especially background clutter and illumination variation, obtaining a significant success overlap score of 0.740/0.742, respectively. We attribute the outstanding performance of the proposed DaDRT tracker to two reasons. Firstly, distractor-aware loss not only effectively alleviates the data-imbalance issue, but also facilitates the robust model update. By automatically mining the most relevant distractor, the updated tracker is robust to the target appearance’s obvious changes and similar backgrounds, which often confuse the existing trackers such as DAT and C-COT. Secondly, the hierarchy-normalized concatenation scheme integrates multiple convolutional layers to strong feature representation and optimized as a whole that can fully take advantage of end-to-end training across multiple convolutional layers.

#### 5.2.2. Comparison with TC-128

**Figure 7:**
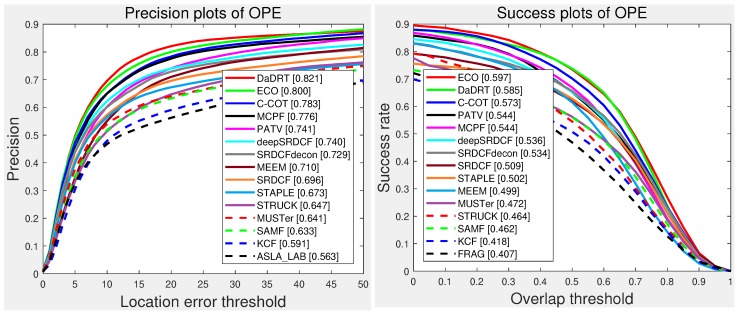
Precision and success plots with the TC-128 dataset using one-pass evaluation. We only show the top 15 performance trackers for representation clarity. Our tracker ranked first in distance precision and second in overlap success. Results are best viewed on high-resolution displays.

The Temple Color 128 (TC-128) dataset contains 128 colorful video sequences. We conducted one-pass evaluation with the same setting as OTB datasets. We evaluated with 18 baseline trackers provided by the authors of the TC-128 and other state-of-the-art trackers, including TLD [81], SAMF [18], MUSTer [68], deepSRDCF [30], SRDCF [22], SRDCFdecon [64], C-COT [31], CREST [38], DSST [19], STAPLE [25], MEEM [67], PATV [74], MCPF [63], and ECO [32]. The OPE precision plots and success plots are shown in Figure 7. Among the evaluated methods, our approach achieved the best distance precision and the second-best AUC success score. The proposed tracker achieved a distance precision score of 0.821, which outperformed the ECO (0.800), MCPF (0.776), and PATV (0.741) methods with a large margin.

#### 5.2.3. Comparison on UAV-123

**Figure 8:**
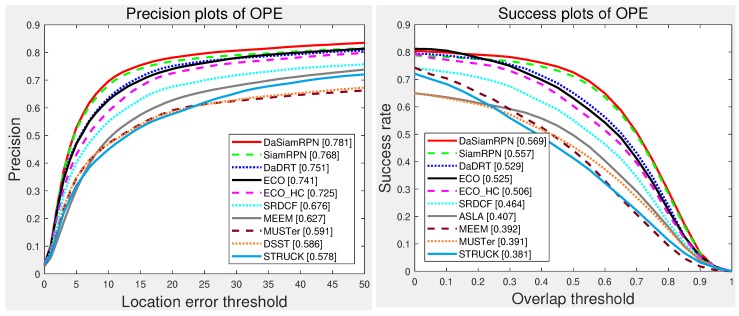
Precision and success plots with the UAV-123 dataset using one-pass evaluation. We only show the top 10 trackers for representation clarity. Our tracker achieved favorable performance (ranking third both in the distance-precision (DP) and area-under-curve (AUC) scores) against state-of-the-art methods. Results are best viewed on high-resolution displays.

The UAV-123 dataset contains 123 video sequences captured from low-altitude unmanned aerial vehicles. Besides the baseline trackers evaluated in the UAV-123 benchmark, we compared the proposed trackers with several representative trackers, including ECO [32], SiamRPN [75], DaSiamRPN [76], and ECO-HC [32]. Figure 8 illustrates the precision and success plots of the compared trackers using one-pass evaluation, respectively. Our approach achieved favorable performance compared with state-of-the-art approaches. Specifically, the performance of the proposed tracker was superior to other regression trackers (e.g., ECO and SRDCF) in terms of distance precision and overlap success score.

#### 5.2.4. Comparison with VOT17

VOT datasets are from the visual-object-tracking (VOT) challenges that provide the tracking community with a precisely defined and repeatable way of comparing short- and long-term trackers, as well as a common platform for discussing the evaluation and advancements made in the field of visual tracking. The standard VOT evaluation scheme applies a reset-based methodology. Whenever a failure (zero overlap with the ground truth) is detected, the tracker is reinitialized five frames after failure. The overall performance is measured by the expected average overlap (EAO), which combines the raw values of per-frame accuracies and failures in a principled manner. Here, we compared the proposed tracker with 51 other state-of-the-art trackers on VOT17 challenges.

**Figure 9:**
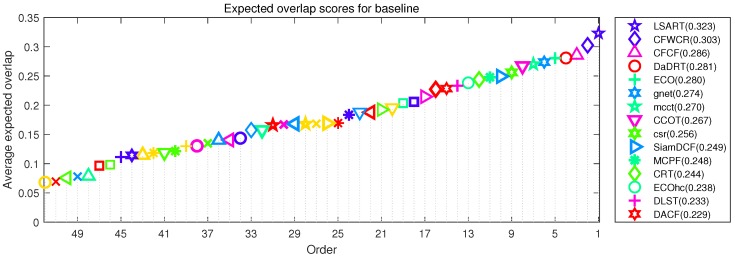
Expected average overlap plot for VOT17 challenge with the proposed Distractor-aware Deep Regression Tracking (DaDRT) tracker. Only the top 15 performing trackers are labeled for clarity. Results are best viewed on high-resolution displays.

Figure 9 illustrates the EAO ranking. Our tracker achieves the remarkable rank (4th). VOT-2017 report [58] recommends a very strict state-of-the-art bound. Any tracker exceeding 0.201 under EAO metric on the VOT17 benchmark is considered the state-of-the-art. The performance of DaDRT is on par with the ECO and better than CCOT, MCPF and CRT. According to the definition of the VOT report, all these trackers are state-of-the-art.

### 5.3. Ablation Studies

**Figure 10:**
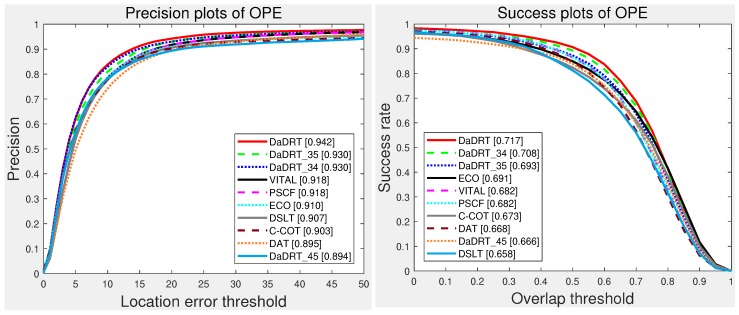
Ablation-study results on the OTB-15 dataset using one-pass evaluation. The numbers in the legend indicate the average distance-precision scores at 20 pixels and the area-under-curve success scores, respectively. Results are best viewed on high-resolution displays.

In DaDRT, we trained the regression network using three convolutional layers and distractor-aware loss. We conducted several experiments to validate the effectiveness of each component. By choosing two of the three used convolutional layers as features, we implemented three alternative approaches to validate multiple convolutional-layer connections. We denoted the three approaches as DaDRT_34, DaDRT_45, and DaDRT_35, where numbers indicate the used convolutional layers. Figure 10 shows the evaluation results on the OTB-15 dataset. We observe that only taking two convolutional layers as features could also achieve great performance compared with other state-of-the-art trackers. Furthermore, the combination across low- and high-level abstractions achieves better accuracy. The performance of DaDRT_34 and DaDRT_35 was almost the same, and the performance of DaDRT_45 was far behind. The evaluation results indicate that spatial detail is more important than semantic abstraction in learning regression networks.

**Figure 11:**
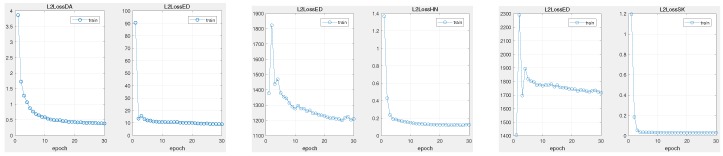
Training progress on different data-imbalance schemes. Each pair illustrates strategy loss and real Euclidean loss. With the same training conditions, all scheme losses rapidly converge. However, the HN and SK losses emerge with large displacement in ED loss. The training process actually requires more training epochs to prevent model overfitting. The proposed distractor-aware loss method can converge rapidly without overfitting. Results are best viewed on high-resolution displays.

Keeping the proposed regression network bone unchanged, we exploited two other data-imbalance schemes, hard-negative mining loss (HN) [41] and shrinkage loss (SK) [39], to exhibit the attribution comparing with other strategies to some extent. In order to obtain intuitive observations, we measured the origin Euclidean distance (ED) between network output and ground truth. The experimental results on sequence bolt are illustrated in Figure 11. From the training progress, we observed that both schemes could handle the data-imbalance issue well, with a fast convergence speed. However, HN and SK both have a large displacement (exceeding 1000) with the ground truth under the same training conditions. Furthermore, our approach can achieve lightweight (less than 20) Euclidean loss. Reviewing the schemes, both HN and SK loss take an importance factor to highlight the valuable rare positive samples and extremely suppress the background, which may make the model overfit to the target. Otherwise, the proposed distractor-aware loss scheme could exploit the distractors from the background context to resist the target without overfitting.

### 5.4. Qualitative Evaluation

**Figure 12:**
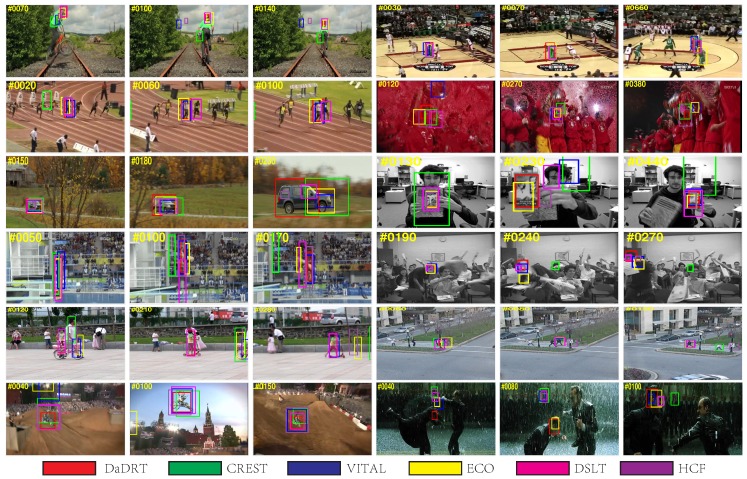
Qualitative results comparing DaDRT with other trackers (ECO, VITAL, CREST, DSLT, HCF) on 12 challenging sequences (from left to right and top to down: *bike*, *basketball*, *bolt2*, *soccer*, *carscale*, *clifbar*, *diving*, *freeman4*, *girl2*, *human3*, *motorrolling and matrix*, respectively). The proposed DaDRT tracker performed favorably against state-of-the-art methods. In the very challenging *soccer*, *clifbar*, and *motorrolling* sequences, DaDRT can always track to the target when most other trackers fail. Results are best viewed on high-resolution displays.

Figure 12 shows qualitative results of the top performing trackers: ECO [32], VITAL [42], CREST [38], DSLT [39], HCF [69] and the proposed DaDRT tracker on 12 challenging sequences. In a majority of these sequences, the CREST tracker failed to locate target objects or incorrectly estimated the scale because of the data-imbalance and numerical issues. The DSLT tracker exploits shrinkage loss to alleviate the data-imbalance issue. The VITAL tracker exploits an adversarial learning scheme to make the model focus on the most temporally robust features, and leverages cost-sensitive loss to handle the data imbalance-issue. The ECO tracker is a correlation-filter-based tracker. It extracts CNN features in addition to handcrafted features (HOG) and independently learns correlation filters. It does not take advantage of information across different features. The proposed DaDRT tracker emphasizes distractors during the training process, which can facilitate the robustness. We further utilized a hierarchy-normalized concatenation connection to fuse abstractions from multiple convolutional layers. The proposed DaDRT tracker performs favorably against state-of-the-art trackers in a majority of challenging sequences.

### 5.5. Failure Case

**Figure 13:**
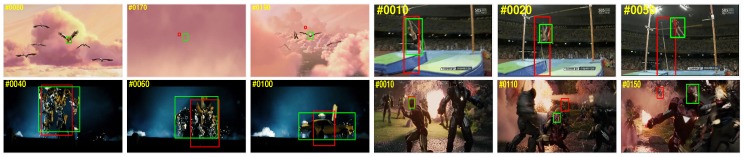
Failure cases of the proposed method on *bird1, jump, trans, ironman* [2], where we used red and green bounding boxes to denote our results and ground truths, respectively. Results are best viewed on high-resolution displays.

We show some typical failure cases of the proposed tracker in Figure 13. The proposed method failed in these cases mainly because of long-term occlusions or nonrigid deformation with scale change. The incremental frame-by-frame model update scheme may draft to the occlusions when long-term occlusions occur (e.g., *bird1*). On the other hand, for nonrigid deformation with scale change (e.g., jump,trans,ironman), the model must take a significant learning to account for large appearance changes. The proposed method balances this dilemma with an asuassive model update pace, which cannot take care of different challenges at the same time. Considering an effective discriminative strategy to distinguishthe different situations may help to alleviate this problem to some extent.

## 6. Conclusions

In this paper, we proposed novel distractor-aware loss to alleviate the data-imbalance issue in learning regression networks. This enables the model to focus on positive and hard-negative samples during the training process. We also applied a hierarchy-normalized concatenation scheme to improve regression learning by exploiting strong representation across multiple convolutional layers. The proposed regression network is fully differentiable and can be trained end to end. Furthermore, incorporating other types of features is straightforward. Extensive experiments on five benchmarks demonstrate that the proposed DaDRT tracker performs favorably against state-of-the-art methods.
